# Effects of Qi-Fu-Yin on aging of APP/PS1 transgenic mice by regulating the intestinal microbiome

**DOI:** 10.3389/fcimb.2022.1048513

**Published:** 2023-01-13

**Authors:** Qiu-yue Xiao, Tian-yuan Ye, Xiao-long Wang, Dong-mei Qi, Xiao-rui Cheng

**Affiliations:** ^1^ Innovative Institute of Chinese Medicine and Pharmacy, Shandong University of Traditional Chinese Medicine, Jinan, China; ^2^ Experimental Center, Shandong University of Traditional Chinese Medicine, Jinan, China

**Keywords:** Qi-Fu-Yin, Alzheimer disease, microbiome, aging, APP/PS1 transgenic mice

## Abstract

**Introduction:**

Alzheimer’s disease is the most common form of dementia and closely related to aging. Qi-Fu-Yin is widely used to treat dementia, but its anti-aging effects is unknown.

**Methods:**

We used 11-month-old APP/PS1 transgenic mice for behavioral tests to observe the changes in cognitive function and age-related symptoms after Qi-Fu-Yin treatment. Fecal samples were collected for 16sRNA sequencing and metagenomic sequencing. Differences among the groups of intestinal microbiota and the associations with aging and intestinal microbiota were analyzed based on the results.

**Results:**

Here we found that Qi-Fu-Yin improved the ability of motor coordination, raised survival rate and prolonged the survival days under cold stress stimulation in aged APP/ PS1 transgenic mice. Our data from 16sRNA and metagenomic sequencing showed that at the Family level, the intestinal microbiota was significantly different among wild-type mice, APP/PS1 transgenic mice and the Qi-Fu-Yin group by PCA analysis. Importantly, Qi-Fu-Yin improved the functional diversity of the major KEGG pathways, carbohydrate-active enzymes, and major virulence factors in the intestinal flora of APP/PS1 transgenic mice. Among them, the functions of eight carbohydrate-active enzymes (GT2_Glycos_transf_2, GT4, GT41, GH2, CE1, CE10, CE3, and GH24) and the functions of top three virulence factors (defensive virulence factors, offensive virulence factors and nonspecific virulence factors) were significantly and positively correlated with the level of grasping ability. We further indicated that the Qi-Fu-Yin significantly reduced the plasma levels of IL-6.

**Conclusion:**

Our results indicated that the effects of Qi-Fu-Yin anti-aging of APP/PS1 transgenic mice might be through the regulation of intestinal flora diversity, species richness and the function of major active enzymes.

## Introduction

Alzheimer’s disease (AD) is one of the most common diseases in the elderly and is the most predominant type of dementia, with clinical manifestations of progressive memory loss and cognitive impairment (Atri, 2019; Scheltens et al., 2021). Although we have more and more understanding of the complex pathogenesis of AD, there are still no definitive conclusions and no disease-modifying treatments (Miller et al., 2011; Weller and Budson, 2018).

Age is the most important factor influencing the onset of AD, and the incidence of AD increases with age (Breijyeh and Karaman, 2020; Qiu et al., 2009). Metabolites of the gut flora as well as pathogenic infections may increase the risk of developing AD (Panza et al., 2019). There is also substantial evidence that microorganisms play an important role in AD (Sorboni et al., 2022). It has also been suggested that the disease may originate in the gut and that abnormal microorganisms affect brain function through different pathways, thus causing neurodegenerative symptoms (Spielman et al., 2018; Cui et al., 2018). For example, intestinal bacteria affect the regulation of the immune system, thus altering the interaction between the immune system and the nervous system (Friedland, 2015; Solas et al., 2017). In addition, the gut microbiota produces metabolites, particularly some short-chain fatty acids with neuroprotective and anti-inflammatory properties, that directly or indirectly affect brain function (Chen et al., 2022). And a study (Abate et al., 2017) have shown that an unhealthy diet can also lead to the formation of toxins, including advanced glycosylation end products (AGEs). These harmful compounds are adsorbed at intestinal levels and can contribute to the aging process. Harmful changes in the aging process may lead to an inflammatory response that further exacerbates neurodegenerative diseases and many other chronic disorders (Hoffman et al., 2017). In contrast, rebuilding healthy flora through diet (Bonfili et al., 2021), probiotics (Den et al., 2020) and prebiotics (Paiva et al., 2020) may promote neural growth, modulate neuroimmunity, and mitigate or even reverse the disease (Pistollato et al., 2018; Shabbir et al., 2021).

APP/PS1 transgenic mice are double transgenic mice expressing human amyloid precursor protein (Mo/HuAPP_695swe_) and mutant human progerin (PS1-dE9), both mutations associated with early-onset AD (Radde et al., 2006). APP/PS1 transgenic mice begins to form amyloid plaques in the brain with impairment in spatial learning memory behavior at 6-7 months of age (Reiserer et al., 2007). However, the degree of memory impairments reached a plateau at 12 months (Zhu et al., 2017). The motility and limb strength of 11-month-old APP/PS1 transgenic mice were significantly aged (Ferguson et al., 2013), possibly corresponding to moderate to severe AD.

More and more studies have shown that traditional Chinese medicine has the effect of preventing and treating dementia (Chen et al., 2019; Wang et al., 2020; Fang et al., 2020), and many small molecules that effectively inhibit AD have been extracted from Chinese herbs (Hyde et al., 2017). Early Clinical studies have shown that tablet huperzine-A can significantly improve cognitive memory in AD patients (Xu et al., 1995). A new clinical study showed that the combination of Dengzhan Shengmai capsule combined with donepezil hydrochloride can improve cognitive function and life capacity in patients with Alzheimer’s disease (Huang et al., 2021). A randomized controlled trial also showed that acupuncture was effective in improving cognitive function and the overall clinical status of AD (Jia et al., 2017). Sodium oligomannate (GV-971), a marine-derived oligosaccharide, is a novel agent that may improve cognition in AD patients (Xiao et al., 2021). Ginkgo biloba extract also has neuroprotective and antioxidant effects on AD and other nervous system diseases (Singh et al., 2019).

In traditional Chinese medicine (TCM) clinics, the initial stage of AD is often characterized by gradual emptying of the medulla oblongata, deficiency of both spleen and kidney, and deficiency of qi and blood (Chen et al., 2020; Li et al., 2021). The Qi-Fu-Yin derived from “Jingyue Quanshu” comprising *Ginseng*, *Rehmannia glutinosa*, *Angelicae Sinensis*, *Atractylodes macrocephala*, *Polygala tenuifolia*, *Glycyrrhiza* and *Spina Date Seed* (Ong et al., 2018). It has the effect of tonifying qi and blood, strengthening the spleen and tranquilizing the mind, coinciding with the heart, spleen, kidney, qi, blood and brain as one treatment for senile dementia (Wang et al., 2014; Ong et al., 2018). In the theory of TCM, Qi is the most basic substance that constitutes and maintains human life activities like vital energy, as well as the driving force that stimulates and regulates human life activities (WuLi et al., 2021). A clinical study showed that the total effective rate of treatment of AD with flavored Qi-Fu-Yin reached 84.38% (Jianming, 2002). Moreover, Qi-Fu-Yin combined with memantine treatment has significant effect on patients with moderate to severe AD (Li, 2020). A meta-analysis showed that Qi-Fu-Yin alone or combination with chemical medicine treatments significantly improved cognitive performance compared to only chemical medicine treatment for dementia (Wang et al., 2021). And Qi-Fu-Yin can alleviate the AD-like pathology and learning memory in model rats through RAGE/NF-κB pathway (Wang et al., 2014). However, it is not known whether Qi-Fu-Yin has any effect on aging in AD.

To investigate whether Qi-Fu-Yin has anti-aging effect in AD, we used 11-month-old APP/PS1 transgenic mice to observe the changes of cognitive function and age-related symptom, after receiving Qi-Fu-Yin treatment.

## Methods

### Preparation of Qi-Fu-Yin

*Ginseng*, *Rehmannia glutinosa*, *Angelicae Sinensis*, *Atractylodes macrocephala*, *Polygala tenuifolia*, *Glycyrrhiza* (Prepared) and *Spina Date Seed* were purchased from Jinan Da Zhai Men Chinese Medicine Co. (Shandong, China) as raw material for the preparation of Qi-Fu-Yin. The ingredients were decocted according to the proportions, and the prepared Qi-Fu-Yin decoction was filtered through four layers of gauze and concentrated to about 2 g/mL.

### Animals and drug treatment

C57BL/6J and PrP-hAβPPswe/PS1ΔE9 (APP/PS1) transgenic mice were purchased from Shanghai Southern Model Laboratory Animal Co., LTD (SCXK (Shanghai) 2017-0010), China. The mice were maintained in the SPF barrier environment at the Experimental Center of Shandong University of Traditional Chinese Medicine under standard housing conditions (room temperature 22 ± 2°C and humidity of 55 ± 10%) with a 12-h light/12-h dark cycle and were allowed free access to water and food.

**Figure 1:**
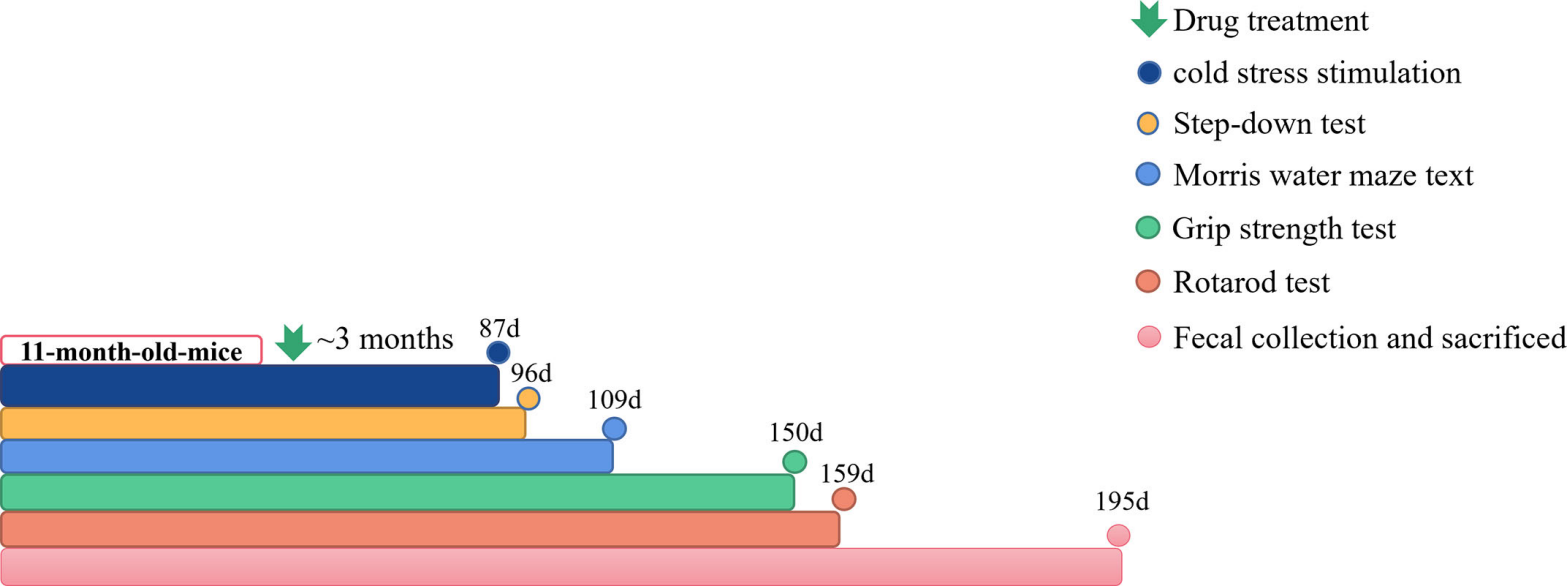
The workflow of animals and drug administration. Cold stress stimulation: n=3~8; Step-down test: n=3~8; Morris water Maze test: n=3~8; Grip strength test: n=3~8; Rotarod test: n=3~8; Fecal collection and sacrificed: n=2~5.

The 11-month-old male C57BL/6J and PrP-hAβPPswe/PS1ΔE9 mice were separated into 6 groups at random, each group had 7-15 mice. APP/PS1 transgenic mice were administrated with Qi-Fu-Yin at 3.1 g/kg/day (L), 6.2 g/kg/day (M), 12.4 g/kg/day (H) for 195 days. APP/PS1 transgenic mice were administrated with Donepezil (1.0mg/kg/d) +Memantine (2.8mg/kg/d) as positive controls (Pos), and C57BL/6J mice as negative controls (Con) and APP/PS1 transgenic mice as AD model (Mod) were given with equal volume of distilled water respectively. After administrating Qi-Fu-Yin for 3 consecutive months, we conducted step-down test, morris water maze test, grip strength test and rotarod test one by one (**Figure 1**). Following the behavioral experiments, plasma was collected for cytokines testing and the stool was collected for metagenomic analysis. The animal treatment, husbandry and experimental protocols in this study were approved (NO. LLSH2019000019) by the Institute of Animal Care and Use Committee (IACUC) of Shandong University of Traditional Chinese Medicine.

### Step-down test

After administrating Qi-Fu-Yin for 96 consecutive days, animals were used to conduct step-down test. The apparatus was a box with a platform (4 cm × 3 cm × 2 cm) in the center of the grid floor. The floor consisted of parallel stainless steel bars. Before training, mice were gently placed in the box to become accustomed to the environment for 3 min. If the mouse stepped down from the wooden platform, they were exposed to an electric foot shock (60-80V, 0.8-1.5mA), and the mice would jump onto the platform to avoid the electric stroke which were maintained for 5 min. Same time at the next day, mice were again placed on the platform, and the time for the animal to step down on the grid floor with all four paws for the first time (latency) and the daily success rate of animals that did not step down.

### Morris water maze test

After administrating Qi-Fu-Yin for 109 consecutive days, Morris water maze test was conducted to evaluated spatial learning and memory ability of mice. This behavioral task included hidden-platform training (spatial learning) and probe trial (spatial memory) session. In the hidden-platform training session, the mice were allowed 4 daily trials in the presence of the platform for 5 subsequent days. In this session, mice were devoted into the pool facing the wall in one of the four quadrants. When the mice located the platform, it was allowed to stay on the platform for 10 s, and if the mice did not locate within 90 s, it was placed on the platform for 10 s to familiarize the environment. The platform was removed after the training experiment, and the mice were placed from the water entry point in the diagonal quadrant where the platform was located and allowed to swim to search it for 90 s. During the whole Morris water maze test, the latency of escape (the time taken to find the hidden platform) in hidden-platform training session, the latency of escape (the first time that the mice crossed the removed platform), the number of crossing the target quadrant and the time in the target quadrant in probe trial session were recorded.

### Grip strength test

After administrating Qi-Fu-Yin for 150 consecutive days, grip strength test was conducted to evaluated grip strength ability of mice. The grip strength measuring instrument was placed horizontally, the mice grip force plate was moved horizontally, and after the mice gripped the grip force plate, it was pulled back with uniform force, causing the mice to release its paw, and the measurement was taken three times continuously.

### Rotarod test

After administrating Qi-Fu-Yin for 159 consecutive days, rotarod test was conducted to evaluate the ability of the limb strength of mice. One day before the experiment, mice were placed on the baton swivel apparatus for regular baton swivel training. The mice were placed into the baton swivel track of the baton swivel apparatus, and the mice were acclimated on the baton for 30 seconds at a speed of 10 r/min, and each was trained for 3 min. In the formal experiment, the mice that had been trained with the regular baton were placed on the baton spinner with a constant speed of 15r/min, and the time that the mice stayed on the baton spinner was recorded, and the duration of the baton spinner was used as an index to measure the motor function of the mice. Dropping or grasping the baton and following it for three times was considered as stopping, and the duration of each rotation was 300 seconds. The test was performed three times consecutively with an interval of 30 min each time, and the final results were averaged from the three experiments.

### Sample collection and DNA extraction

Approximately 200 mg of fresh feces were collected from each mouse, and all fecal specimens were freely defecated by the mice and collected immediately. Among the 12 mice fecal samples analyzed by sequencing, 5 were from the control group, 2 were from the model group, and 5 were from the high-dose group of Qi-Fu-Yin. Total genomic DNA (gDNA) of every mice’s fresh stool was isolated using the E.Z.N.A.^®^ soil DNA Kit (Omega Bio-tek, Norcross, GA, U.S.) according to the manufacturer’s protocol. The quality of gDNA was confirmed using an agarose gel and the final gDNA yield was quantified using the NanoDrop2000 (Thermo, USA).

### 16S rRNA amplicon sequencing of gut microbiota

Universal primers 338F (5’- ACTCCTACGGGAGGCAGCAG-3’) and 806R (5’- GGACTACHVGGGTWTCTAAT-3’) were employed to amplify gDNA. Purified amplicons were pooled in equimolar and paired-end sequenced on an Illumina MiSeq PE300 platform (Illumina, San Diego, USA) according to the standard protocols. The raw 16S rRNA gene sequencing reads were demultiplexed, quality-filtered by FASTp software (https://github.com/OpenGene/fastp, version 0.20.0) and merged by FLASH software (http://www.cbcb.umd.edu/software/flash, version 1.2.7). Operational taxonomic units (OTUs) with 97% similarity cutoff were clustered using UPARSE (http://drive5.com/uparse/, version 7.1), and chimeric sequences were identified and removed. The taxonomy of each OTU representative sequence was analyzed by RDP Classifier (http://rdp.cme.msu.edu/, version 2.2) against the 16S rRNA database using confidence threshold of 70%.

### Library construction and metagenomic sequencing

DNA extract was fragmented to an average size of about 400 bp using Covaris M220 (Gene Company Limited, China) for paired-end library construction. Paired-end library was constructed using NEXTFLEX^®^ Rapid DNA-Seq (Bioo Scientific, Austin, TX, USA). Adapters containing the full complement of sequencing primer hybridization sites were ligated to the blunt-end of fragments. Paired-end sequencing was performed on Illumina NovaSeq (Illumina Inc., San Diego, CA, USA) at Majorbio Bio-Pharm Technology Co., Ltd. (Shanghai, China) using NovaSeq Reagent Kits according to the manufacturer’s instructions (www.illumina.com). The data were analyzed on the free online platform of Majorbio Cloud Platform (www.majorbio.com). The paired-end Illumina reads were trimmed of adaptors, and low-quality reads (length<50 bp or with a quality value<20 or having N bases) were removed by FASTp (https://github.com/OpenGene/fastp, version 0.20.0).

### Microbial diversity and functional annotation analysis

Genes with nucleic acid length greater than or equal to 100 bp were selected and translated into amino acid sequences to obtain a statistical table of gene prediction results for each sample. The predicted gene sequences of all samples were clustered (parameters: 90% identity, 90% coverage) using CD-HIT software (http://www.bioinformatics.org/cd-hit/), and the high quality reads of each sample were compared with the non-redundant gene sets (95%identity) using SOAPaligner software (http://soap.genomics.org.cn/, version 2.21), the high quality reads of each sample were compared with the non-redundant gene set separately (95%identity), and the abundance information of genes in the corresponding samples was counted. Rarefaction curves, measured by observed OTU, Shannon index, and alpha diversity index. Representative sequences of non-redundant gene catalog were aligned to NCBI NR database with e-value cutoff of 1e^-5^ using Diamond (http://www.diamondsearch.org/index.php, version 0.8.35) for taxonomic annotations. Cluster of orthologous groups of proteins (COG) annotation for the representative sequences was performed using Diamond against eggNOG database with an e-value cutoff of 1e^-5^. The KEGG annotation was conducted using Diamond against the Kyoto Encyclopedia of Genes and Genomes database (http://www.genome.jp/keeg/) with an e-value cutoff of 1e^-5^. Carbohydrate-active enzymes annotation was conducted using hmmscan (http://hmmer.janelia.org/search/hmmscan) against CAZy database (http://www.cazy.org/) with an e-value cutoff of 1e^-5^. Virulent factor annotation was conducted using Diamond against VFDB database (http://www.mgc.ac.cn/VFs/) with an e-value cutoff of 1e^-5^.

### Luminex multi-factor assay

After 195d administration of Qi-Fu-Yin in 11-month-old APP/PS1 transgenic mouse, the eyeballs were removed for blood samples collected by EP tubes containing sodium heparin, and blood samples were centrifuged at 3500 rpm for 15 min (at room temperature) after 30 min, and the levels of IL-5, IL-6 and TNF-α cytokines in mice plasma were measured using the Luminex Liquid Phase Suspension Chip System according to the experimental procedure in Luminex Assay Mouse Premixed Multi-Analyte Kit (L136639, R&D systems).

### Statistical analysis

Data are expressed as mean ± SEM and analyzed by employing the GraphPad Prism 8.0.1 software. The Mann Whitney non-parametric test and unpaired *t*-test were used to compare genotypes, and Ordinary one-way ANOVA was used to compare differently treated groups. In addition, the area under curve (AUC) was calculated for the learning curves. The statistical analyses used *p*<0.05 as a basis for the level of significance.

## Results

### The effect of Qi-Fu-Yin on cold stress stimulation in APP/PS1 transgenic mice

**Figure 2:**
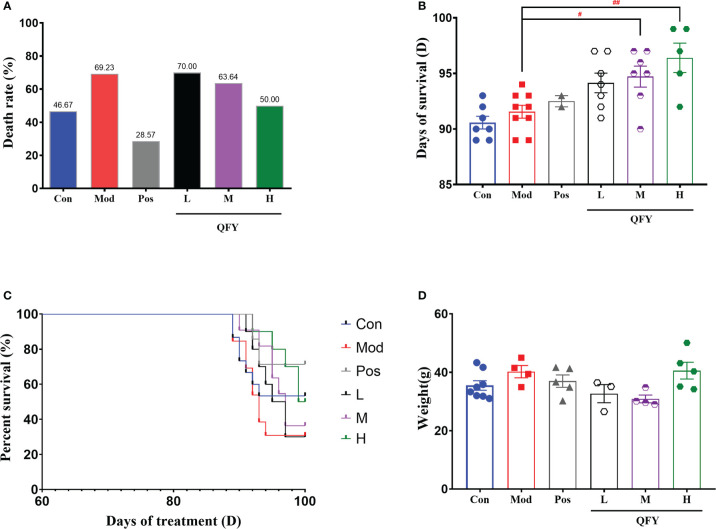
Effects of Qi-Fu-Yin on environmental cold stress-induced mortality in aged APP/PS1 transgenic mice. **(A)** The percent of death, **(B)** Days of treatment on death, **(C)** The percent of survival. **(D)** Weight after cold stress. Mean ± SEM, n=3~8, vs control (Con) group, unpaired Student`s *t*-test; ^#^ *P*<0.05, ^##^ *P*<0.01, vs model (Mod) group, one-way ANOVA analysis followed by Dunnett’s multiple comparisons test, Graphad Prism 8.0.1 software.

On the 87th day of administration to 11-month-old male APP/PS1 transgenic mice, the air conditioner in the animal feeding room malfunctioned, causing the temperature in the feeding room to drop and remain at 19-21°C. After continuing for 3 days, the temperature rose to 23-25°C. From the 89th day of administration, animals in each group started to die successively, and the statistics of the death of the mice are shown in **Table 1**. The death rate of wild-type mice was 46.67% and the overall mortality rate of APP/PS1 transgenic mice was 60% (**Table 1**). The percent of death was 46.67% in the control group, 69.23% in the model group, 28.57% in the positive drug group, 70% in the low-dose group of Qi-Fu-Yin, 63.64% in the middle-dose group of Qi-Fu-Yin, and 50% in the high-dose group of Qi-Fu-Yin (**Table 1**; **Figure 2A**). The above data illustrated that the death rate of APP/PS1 transgenic mice administered with the positive drug and Qi-Fu-Yin was reduced, and there was a certain dose effect on the death rate of mice administered with Qi-Fu-Yin. The higher the dose, the lower the death rate, and the high dose of Qi-Fu-Yin reduced the death rate (19.23% reduction) of APP/PS1 transgenic mice significantly. Compared with the model group, the survival days of APP/PS1 transgenic mice were significantly prolonged (4.8 days on average) in both the middle (*P*<0.05) and high doses (*P*<0.01) of Qi-Fu-Yin (**Figure 2B**). Qi-Fu-Yin could reduce the death rate and prolonged the survival of old APP/PS1 transgenic mice (**Figure 2C**). There was no significant difference in weightbetween the groups (**Figure 2D**). These data suggested that APP/PS1 transgenic mice have a poor ability to cope with environmental changes, especially cold stress, which can be significantly enhanced by Qi-Fu-Yin with some dose effect.

**Table 1:** The effects of Qi-Fu-Yin on survival of aged APP/PS1 transgenic mice induced by environmental cold stress.

Group	Con	Mod	Pos	L	M	H	Sum
Sum (number)	15	13	7	10	11	10	67
Death (number)	7	9	2	7	7	5	38
Survival (number)	8	4	5	3	4	5	29
The rate of death (%)	46.67	69.23	28.57	70.00	63.64	50.00	56.06

Con: C57/BL6J or wild type; Mod: APP/PS1 transgenic mice; Pos: Donepezil (1.0mg/kg/d) +Memantine (2.8mg/kg/d); L: APP/PS1 transgenic mice + Qi-Fu-Yin (3.1 g/kg/day); M: APP/PS1 transgenic mice + Qi-Fu-Yin (6.2 g/kg/day); H: APP/PS1 transgenic mice + Qi-Fu-Yin (12.4 g/kg/day).

$\text{ The rate of death}\left(\%\right)=\;\frac{Number\;of\;death\text{s}}{Sum}\times 100\%$ .

$\text{ The rate of survival}\left(\%\right)=\;\frac{Sum-Number\;of\;death\text{s}}{Sum}\times 100\%$ .

### The effects of Qi-Fu-Yin on cognitive impairments of APP/PS1 transgenic mice

**Figure 3:**
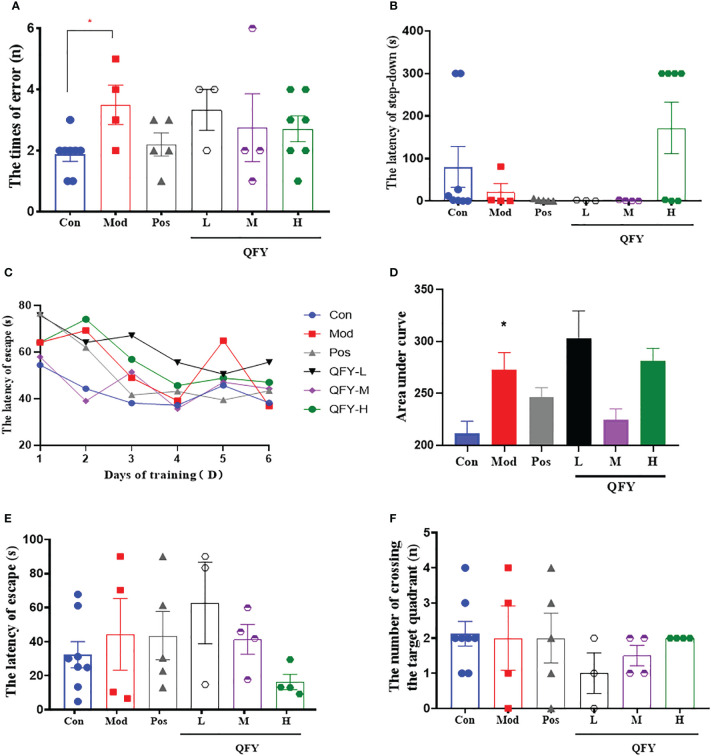
Effects of Qi-Fu-Yin on cognitive impairments in APP/PS1 transgenic mice. Step-down test **(A, B)** and Morris water Maze test **(C–F)**. **(A)** The times of errors during learning period, **(B)** The latency of step-down during test period, **(C)** The latency of escape in hidden-platform training session, **(D)** Area under curve (AUC) for the latency of escape in hidden-platform training session, **(E)** The latency of escape in probe trial session, **(F)** The number of crossing the target quadrant in probe trial session. Mean ± SEM, n=3~8, ^*^ *P*<0.05, vs control (Con) group, unpaired Student`s *t*-test; vs model (Mod) group, one-way ANOVA analysis followed by Dunnett’s multiple comparisons test, Graphpad Prism 8.0.1 software.

After the 11-month-old male APP/PS1 transgenic mice treated by Qi-Fu-Yin for 96 days, Step-down test was employed to measure the ability of passive avoidance response of mice. The results showed the number of errors in the learning period (**Figure 3A**) was significantly increased compared to control group (*P*<0.05), while the treatment of Qi-Fu-Yin in high-dose tended to decreased in APP/PS1 transgenic mice. The latency of step-down in the test period (**Figure 3B**) was tended to shorten compared to control group, while the high-dose of Qi-Fu-Yin tended to prolonged in APP/PS1 transgenic mice. These data indicated that Qi-Fu-Yin might had beneficial effects on the passive avoidance response ability of aged APP/PS1 transgenic mice.

Morris water Maze test was used to detect spatial learning and memory ability of 11-month-old male APP/PS1 transgenic mice after 109 days administration of Qi-Fu-Yin. The results showed that the area under curve (AUC, **Figure 3D**) of latency in learning period was significantly increased in APP/PS1 transgenic mice compared to control group (*P*<0.05), while the middle dose of Qi-Fu-Yin had a tendency to shorten this latency (**Figures 3C, D**) in APP/PS1 transgenic mice, and the high dose of Qi-Fu-Yin had a tendency to shorten the latency period of the probe trial (**Figure 3E**), and had no significant effect on the number of crossing the target quadrant (**Figure 3F**). These results suggested that Qi-Fu-Yin may have a positive effect on the spatial learning memory ability of aged APP/PS1 mice effect.

### The effect of Qi-Fu-Yin on aging in APP/PS1 transgenic mice

**Figure 4:**
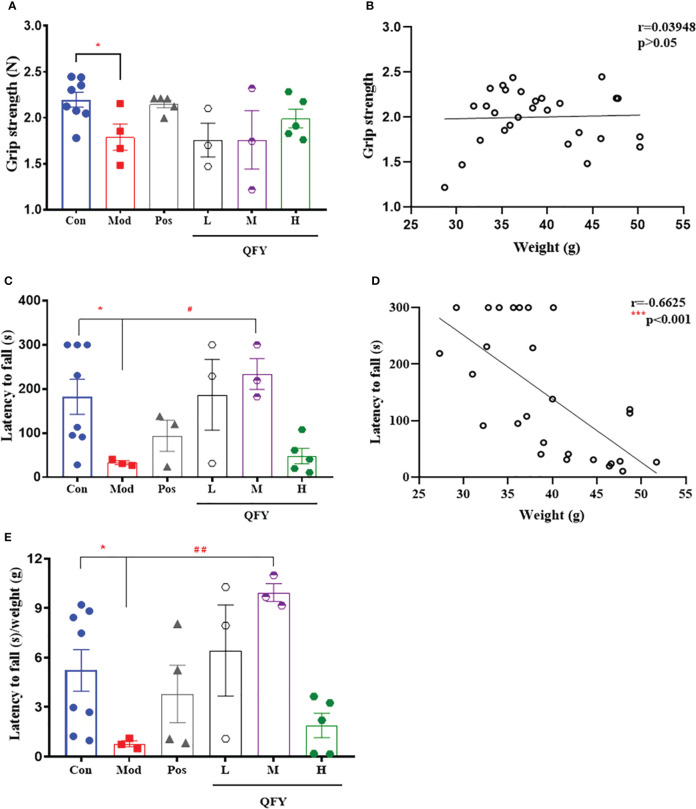
Effects of Qi-Fu-Yin on the ability of grip strength and the limb strength of APP/PS1 transgenic mice. Grip strength test **(A, B)** and Rotarod test **(C–E)**. **(A)** Grip strength, **(B)** The correlation between grip strength and body weight, **(C)** latency to fall, **(D)** The correlation between latency to fall and body weight, **(E)** latency to fall/weight. Mean ± SEM, n=3~8, ^*^ *P*<0.05, vs control (Con) group, unpaired Student`s t-test, ^#^ *P*<0.05, ^##^ *P*<0.01, vs model (Mod) group, one-way ANOVA analysis followed by Dunnett’s multiple comparisons test, ^***^ *P*<0.001, Pearson correlation analysis, Graphpad Prism8.0.1 software.

After the 11-month-old male APP/PS1 transgenic mice treated by Qi-Fu-Yin for 150 days, the grip strength test was employed to measure the grip strength of the forelimbs of mice. The results showed that the grip strength (**Figure 4**) of mice in the APP/PS1 transgenic mice was significantly decreased compared with control group (*P*<0.05), while the high dose of Qi-Fu-Yin had a tendency to enhance the grip strength in APP/PS1 transgenic mice. And there was no significant correlation between grip strength and body weight (**Figure 4B**). These data indicated that Qi-Fu-Yin might had effect on enhancing the limb strength of aged APP/PS1 transgenic mice.

Qi-Fu-Yin was orally administered to 11-month-old male APP/PS1 transgenic mice for 159 days, and the ability of motor coordination in mice was detected by rotarod test. The results showed that the latency to fall (**Figure 4C**) in APP/PS1 transgenic mice was significantly shorter compared with the control group (*P*<0.05), while the middle dose of Qi-Fu-Yin significantly prolonged the latency to fall (*P*<0.05), even after correction using body weight (*P*<0.01) (**Figure 4E**). There was a significant negative correlation (*P*<0.001) between latency to fall and body weight (*P*<0.001) (**Figure 4D**). These results revealed that Qi-Fu-Yin has the effect of significantly enhancing the motor coordination ability of aged APP/PS1 transgenic mice.

### The treatment of Qi-Fu-Yin improved the species diversity of gut microbiota in aged APP/PS1 transgenic mice

In order to observe the effect of Qi-Fu-Yin on life span, the administration was continued after measuring the above indexes. Until 186 days of administration, one of the four mice in the model group died naturally, and the remaining 3 mice began to be tested for bacterial flora. Five of the eight mice in controls and five mice of the high-dose group were each taken, and only two mice of the model group were sequenced because one of the three mice in the model group had diarrhea that prevented collect the stool completely. Feces were collected on 194 days of administration for testing.

**Figure 5:**
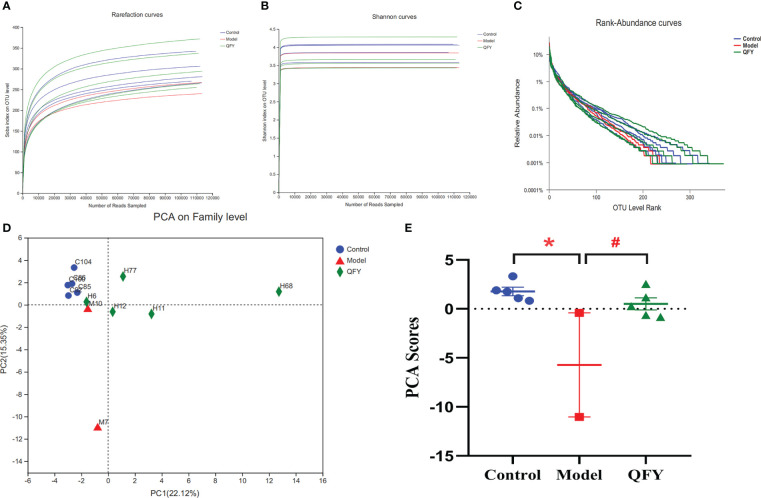
Effects of Qi-Fu-Yin on the number and diversity of gut microbiota species in APP/PS1 transgenic mice. **(A)** Alpha diversity analysis of species abundance, **(B)** Alpha diversity analysis of diversity, **(C)** Rank-Abundance analysis of species richness and community evenness, **(D)** PCA on the Family level, the first two principal coordinates PC1 and PC2 are plotted. **(E)** PCA scores according to the sample coordinates. Mean ± SEM, n=2~5, * *P*<0.05, unpaired Student’s-*t* test with one-tailed. GraphPad Prism 8.0.1 software.

We collected stool samples and performed 16S rRNA amplicon sequencing (16S sequencing) and macrogenome sequencing on gDNA extracted from 12 samples. The sequence information of bacterial 16S rRNA was obtained based on Illumina Miseq sequencing platform. A total of 1422359 valid sequences were obtained from this sequencing (**Table 2**). Among them, the lengths of all sequences were mainly distributed in the interval of 401-440bp. The comparison of alpha diversity indices revealed (**Figures 5A, B**) that the alpha diversity rarefaction curve of samples tended to be flat, indicating that the amount of sequencing data was reasonable. In total of 577 OTUs were detected by quality control and operational taxonomic unit (OTU) clustering. The Rank-Abundance results showed (**Figure 5C**) that the model group had lower species richness and uneven species distribution compared to the control group, while the Qi-Fu-Yin group had a tendency to increase species richness and evenness of species distribution.

**Table 2:** The statistics of effective sequence information were obtained by sequencing.

Samples	Sequences	Bases(bp)	Average Length
12	1422359	598886168	421.0591903

We found that at the classification level of Phylum and below, the number of species in APP/PS1 transgenic mice was lower than that in the control group, while the number of species in Qi-Fu-Yin was similar to the control group as shown in Venn diagrams (**Supplementary Image 1**). These results suggesting that Qi-Fu-Yin has a regulatory effect on intestinal bacteria species in APP/PS1 transgenic mice. The results by Principal Component Analysis (PCA) analysis (**Figure 5D**) showed that the gut microbiota of APP/PS1 transgenic mice administrated with Qi-Fu-Yin and control group were clustered together on the Family level, while the gut microbiota of APP/PS1 transgenic mice group were distinct from the other two groups (**Figure 5E**). It is suggested that the intestinal microbiota of the treatment of Qi-Fu-Yin were more similar to those of the control group than the APP/PS1 transgenic mice group, which indicated that the treatment of Qi-Fu-Yin might have the ability to restore, at least in part, the gut microbial communities of APP/PS1 transgenic mice to those of the C57/BL6J mice.

**Figure 6:**
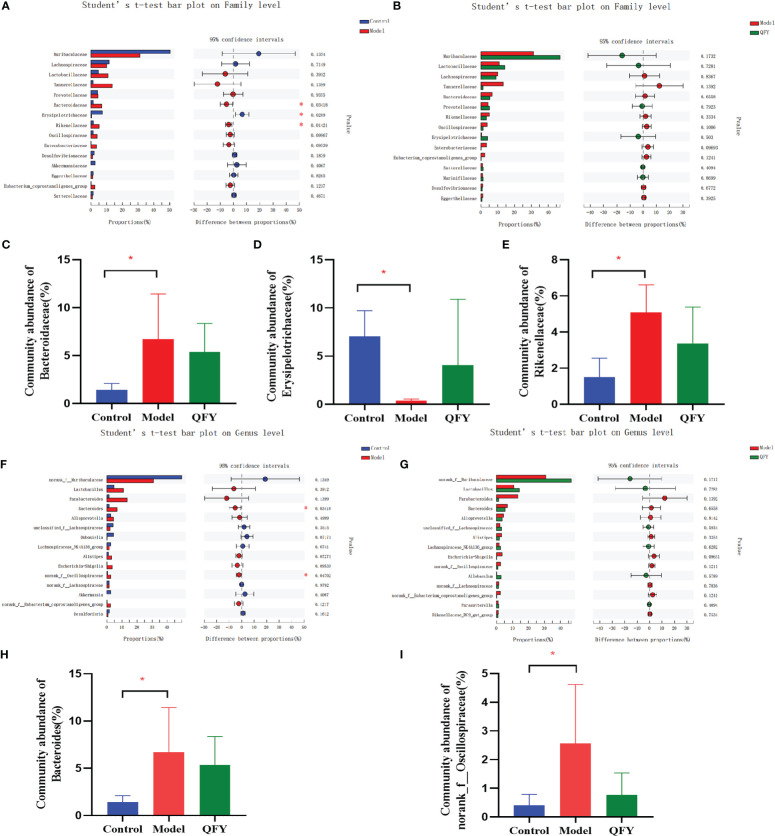
The effect of Qi-Fu-Yin on the relative species abundance of intestinal microorganisms in APP/PS1 transgenic mice. **(A)** Difference between control group and model group on family level, **(B)** Difference between model group and Qi-Fu-Yin group on family level, **(C)** Community abundance of *Bacteroidaceae*, **(D)** Community abundance of *Rikenellaceae*, **(E)** Community abundance of *Erysipelotrichaceae*, **(F)** Difference between control group and model group on genus level, **(G)** Difference between model group and Qi-Fu-Yin group on genus level, **(H)** Community abundance of *Bacteroides*, **(I)** Community abundance of *norank_f:Oscillospiraceae.* Mean ± SD, n=2~5, *P<0.05, unpaired Student’s-*t* test. GraphPad Prism 8.0.1 software.

We found that *Bacteroidetes* had the highest proportion of intestinal flora in all three groups, followed by *Firmicutes*. At the family level (**Figures 6A, B**), the structural composition of the gut microbiota of control group and APP/PS1 transgenic mice was significantly different, and administration of Qi-Fu-Yin to APP/PS1 transgenic mice caused their gut microbiota composition to become similar to that of control mice. Compared with the control group, the abundance of *Bacteroidaceae* (*P*<0.05) (**Figure 6C**) and *Rikenellaceae* (*P*<0.05) (**Figure 6E**) were significantly increased, and the abundance of *Erysipelotrichaceae* (*P*<0.05) (**Figure 6D**) were significantly decreased in APP/PS1 transgenic mice, while Qi-Fu-Yin has a tendency to reverse the abundance of these mycobacterial families. At the genus level (**Figures 6F, G**), the abundance of *Bacteroides* (*P*<0.05) (**Figure 6H**) and *norank_f:Oscillospiraceae* (*P*<0.05) (**Figure 6I**) were significantly increased in APP/PS1 transgenic mice compared with control mice, while there was a tendency to decrease them in APP/PS1 transgenic mice treated with Qi-Fu-Yin. This suggested that Qi-Fu-Yin may improve cognition and aging by regulating the species richness of *Bacteroidaceae*, *Rikenellaceae* and *Erysipelotrichaceae.*

### The treatment of Qi-Fu-Yin improved the functional diversity of gut microbiota in APP/PS1 transgenic mice

Functional profiles of each group of mouse intestinal flora were obtained through three major databases, Evolutionary genealogy of genes: Non-supervised Orthologous Groups (EggNOG), Kyoto Encyclopedia of Genes and Genomes (KEGG) and Carbohydrate-active enzymes (CAZyme) databases. The results showed that the gene annotation of all samples yielded a total of 10678 in EggNOG, 13299 in KEGG and 455 in CAZyme at the Family level.

**Figure 7:**
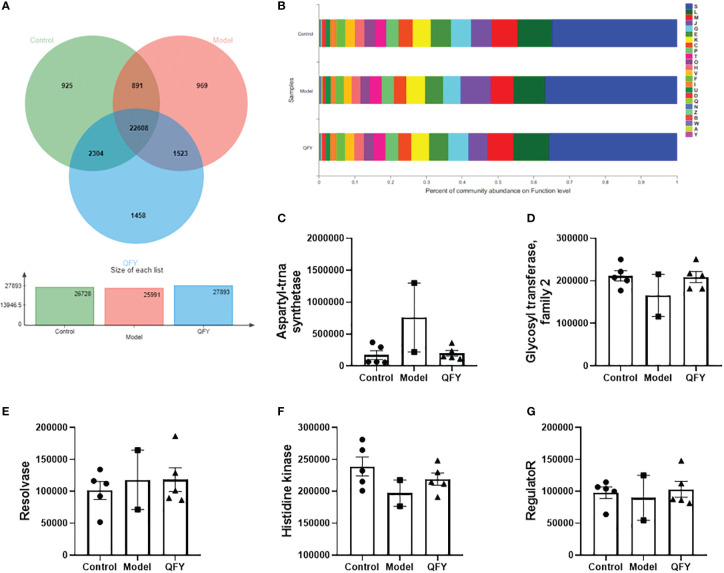
The effect of Qi-Fu-Yin on the function of major COG of intestinal microorganisms in APP/PS1 transgenic mice. **(A)** Functional profiles of each group in EggNOG, **(B)** Percent of community abundance on function level, **(C)** Genes abundance in COG0173 (aspartyl-trna synthetase), **(D)** Genes abundance in COG1961 (Resolvase), **(E)** Genes abundance in COG0463 (Glycosyl transferase, family 2), **(F)** Genes abundance in ENOG410XNMH (Histidine kinase), **(G)** Genes abundance in COG0745 (regulatoR). Mean ± SEM, n=2~5, unpaired Student’s-*t* test. GraphPad Prism 8.0.1 software. A: RNA processing and modification; B: Chromatin structure and dynamics; C: Energy production and conversion; D: Cell cycle control, cell division, chromosome partitioning; E: Amino acid transport and metabolism; F: Nucleotide transport and metabolism; G: Carbohydrate transport and metabolism; H: Coenzyme transport and metabolism; I: Lipid transport and metabolism; J: Translation, ribosomal structure and biogenesis; K: Transcription; L: Replication, recombination and repair; M: Cell wall/membrane/envelope biogenesis; N: Cell motility; O: Post-translational modification, protein turnover, chaperones; P: Inorganic ion transport and metabolism; Q: Secondary metabolites biosynthesis, transport and catabolism; S: Function unknown; T: Signal transduction mechanisms; U: Intracellular trafficking, secretion, and vesicular transport; V: Defense mechanisms; W: Extracellular structures; Y: Nuclear structure; Z: Cytoskeleton.

The distribution of the main functions of the mouse intestinal flora in each group was observed in the EggNOG database. The total number of functional profiles in EggNOG in the three groups was 22,608, of which 925 were unique to C57 mice, 969 were unique to APP/PS1 transgenic mice, and 1,458 were unique to the Qi-Fu-Yin group (**Figure 7A**). The results showed (**Figure 7B**) that among the known functions, the dominant functions (relative abundance >1%) of the mouse intestinal flora in each group were mainly distributed among 24 Functions, of which the top 5 Functions were L (Replication, recombination and repair), M (Cell wall/membrane/envelope biogenesis), G (Carbohydrate transport and metabolism), E (Amino acid transport and metabolism), K (Transcription), and about 40% of the unknown functions S.

The sequences of non-redundant gene sets were compared with the eggNOG database to obtain the Clusters of orthologous groups of proteins (COG) corresponding to the genes. The comparison of the COG functions of each group showed (**Figures 7C–G**) that among the top 5, the gene abundance of COG0173 (aspartyl-trna synthetase) and COG1961 (Resolvase) in the intestinal flora of APP/PS1 transgenic mice tended to increase compared with control mice, while the gene abundance of aspartyl-trna synthetase tended to decrease with Qi-Fu-Yin. The gene abundance of COG0463 (Glycosyl transferase, family 2), ENOG410XNMH (Histidine kinase) and COG0745 (regulatoR) in the intestinal flora of APP/PS1 transgenic mice tended to decrease compared with control mice, while the genes abundance of Qi-Fu-Yin tended to increase.

The top 5 Functions in the intestinal flora of control mice were Histidine kinase; Glycosyl transferase, family 2; aspartyl-trna synthetase; Mate efflux family protein; hydrolase family 2. The top 5 Functions in the intestinal flora of APP/PS1 transgenic mice were aspartyl-trna synthetase; Histidine kinase; Glycosyl transferase, family 2; Mate efflux family protein; TonB dependent receptor, and the top 5 Functions in the intestinal flora of Qi-Fu-Yin mice were Histidine kinase; Glycosyl transferase, family 2; aspartyl-trna synthetase; Mate efflux family protein; TonB dependent receptor. The results showed that aspartyl-trna synthetase was the main adverse function group activated in APP/PS1 transgenic mice compared with the control group, and Qi-Fu-Yin had an inhibitory effect. APP/PS1 transgenic mice may have increased the behavioral disorder by activating aspartyl-trna synthetase, while Qi-Fu-Yin can improve this impairment.

**Figure 8:**
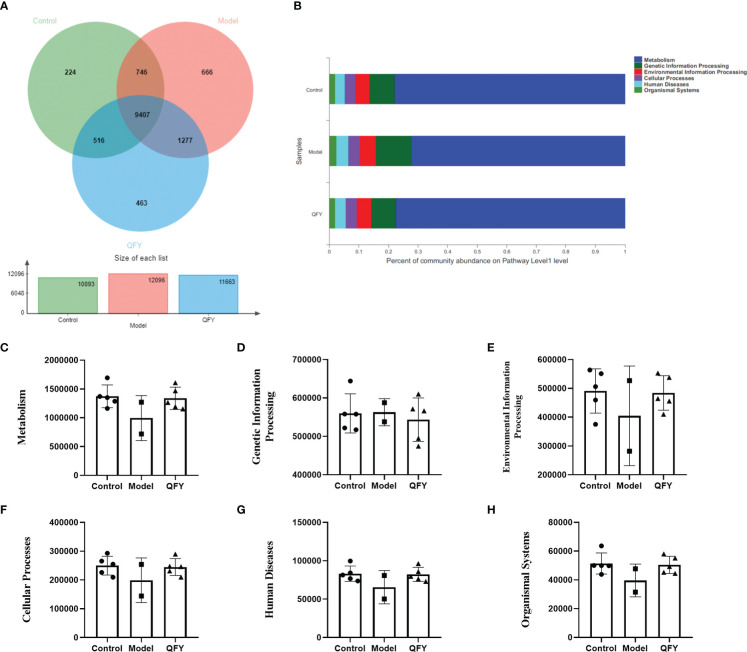
The effect of Qi-Fu-Yin on the function of KEGG of intestinal microorganisms in APP/PS1 transgenic mice **(A)** Functional profiles of each group in KEGG, **(B)** Percent of community abundance on pathway level 1, **(C)** Genes abundance in Metabolism, **(D)** Genes abundance in Genetic Information Processing, **(E)** Genes abundance in Environmental Information Processing, **(F)** Genes abundance in Cellular Processes, **(G)** Genes abundance in Human Diseases, **(H)** Genes abundance in Organismal Systems. Mean ± SD, n=2~5, unpaired Student’s-*t* test. GraphPad Prism 8.0.1 software.

The distribution of the main functions of the intestinal flora of each group of mice was observed at the Pathway level in the KEGG database. The total number of functional profiles in KEGG in the three groups was 9407, of which 224 were unique to C57 mice, 666 were unique to APP/PS1 transgenic mice, and 463 were unique to the Qi-Fu-Yin group (**Figure 8A**). The results indicated (**Figure 8B**) that the Pathway functions were mainly divided into six main categories, Metabolism, Genetic Information Processing, Environmental Information Processing, Cellular Processes, Human Diseases, and Organismal Systems. The differences in KEGG function at pathway level were compared among the groups. The results showed that there was a trend of decreasing the abundance of all five KEGG genes in the intestinal flora of APP/PS1 transgenic mice for metabolism (**Figure 8C**), environmental information processing (**Figure 8E**), cellular processes (**Figure 8F**), human diseases (**Figure 8G**), and organismal systems (**Figure 8H**) compared with control group, while Qi-Fu-Yin had a tendency to elevate them. Qi-Fu-Yin may play a role in anti-aging and cognitive improvement by regulating metabolism, cellular processes and other pathways.

**Figure 9:**
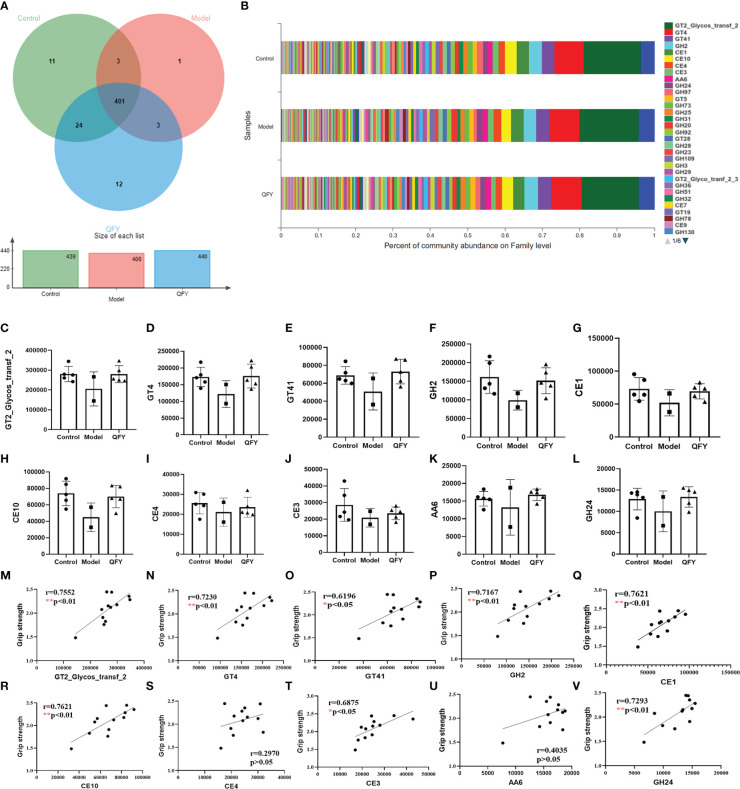
The effect of Qi-Fu-Yin on the function of CAZy of intestinal microorganisms in APP/PS1 transgenic mice. **(A)** Functional profiles of each group in CAZy (family), **(B)** Percent of community abundance on family level, **(C)** GT2_Glycos_transf_2 (Glycosyl Transferases), **(D)** GT4 (Glycosyl Transferases), **(E)** GT41 (Glycosyl Transferases), **(F)** GH2 (Glycoside Hydrolases), **(G)** CE1 (Carbohydrate Esterases), **(H)** CE10 (Carbohydrate Esterases), **(I)** CE4 (Carbohydrate Esterases), **(J)** CE3 (Carbohydrate Esterases), **(K)** AA6 (Auxiliary Activities), **(L)** GH24 (Glycoside Hydrolases), **(M)** The correlation between grip strength and abundance of GT2_Glycos_transf_2, **(N)** The correlation between grip strength and abundance of GT4, **(O)** The correlation between grip strength and abundance of GT41, **(P)** The correlation between grip strength and abundance of GH2, **(Q)** The correlation between grip strength and abundance of CE1, **(R)** The correlation between grip strength and abundance of CE10, **(S)** The correlation between grip strength and abundance of CE4, **(T)** The correlation between grip strength and abundance of CE3, **(U)** The correlation between grip strength and abundance of AA6, **(V)** The correlation between grip strength and abundance of GH24. Mean ± SD, n=2~5, ^*^ *P* < 0.05, ^**^ *P* < 0.01, unpaired Student’s-*t* test. GraphPad Prism 8.0.1 software.

The total number of functional profiles in the three groups of CAZyme was 455 (**Supplementary Table 1**), of which 11 were unique to C57 mice, one to APP/PS1 transgenic mice, and 12 to the Qi-Fu-Yin group (**Figure 9A**). Among the CAZy obtained from Family level annotation, the top 10 CAZy (**Figure 9B**) in order of abundance in the total sample were four carbohydrate esterases (CE) CE1, CE10, CE4, CE3, three glycosyltransferases (GT) GT2_Glycos_transf_2, GT4 and GT41, one glycoside hydrolase GH2, one reductase AA6, and a lysozyme GH24. Compared with control mice, there was a tendency to decrease the genes abundance of the top 10 (GT2_Glycos_transf_2, GT4, GT41, GH2, CE1, CE10, CE4, CE3, AA6, and GH24) CAZy (**Figures 9C–L**) in the intestinal flora of APP/PS1 transgenic mice, while there was a tendency to elevate them in administration of Qi-Fu-Yin. There was a significant positive correlation between grip strength and GT2_Glycos_transf_2 (*P*<0.01) (**Figure 9M**), GT4 (*P*<0.01) (**Figure 9N**), GT41 (*P*<0.05) (**Figure 9O**), GH2 (*P*<0.01) (**Figure 9P**), CE1 (*P*<0.01) (**Figure 9Q**), CE10 (*P*<0.01) (**Figure 9R**), CE3 (*P*<0.05) (**Figure 9T**), and GH24 (*P*<0.01) (**Figure 9V**), and no significant correlation between grip strength and CE4 (**Figure 9S**, AA6 (**Figure 9U**). These results suggested that the above active enzymes may have a positive regulatory effect on grip strength in mice, and Qi-Fu-Yin may improve the motor coordination ability of aged mice by regulating related active enzymes.

**Figure 10:**
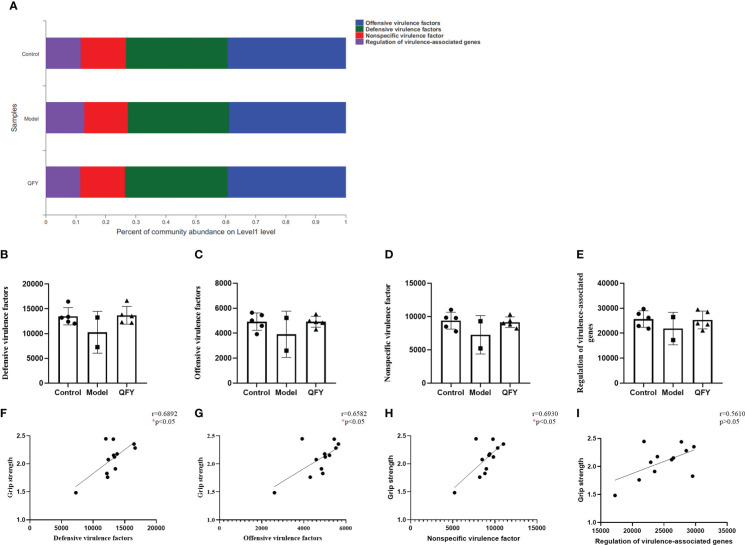
The effect of Qi-Fu-Yin on the function of virulence factor of intestinal microorganisms in APP/PS1 transgenic mice. **(A)** Percent of community abundance on virulence factor level 1 **(B)** Defensive virulence factors, **(C)** Offensive virulence factors, **(D)** Nonspecific virulence factor, **(E)** Regulation of virulence-associated genes, **(F)** The correlation between grip strength and abundance of defensive virulence factors, **(G)** The correlation between grip strength and abundance of offensive virulence factors, **(H)** The correlation between grip strength and abundance of nonspecific virulence factor, **(I)** The correlation between grip strength and abundance of regulation of virulence-associated genes. Mean ± SD, n=2~5, ^*^ *P* < 0.05, unpaired Student’s-*t* test. GraphPad Prism 8.0.1 software.

Based on the virulence factor database (VFDB, http://www.mgc.ac.cn/VFs/), we observed the functional distribution of the main virulent factors in the intestinal flora of each group of mice, and the results showed (**Figure 10A**) that the virulence factors were divided into 4 main categories, Offensive virulence factors, Defensive virulence factors, Nonspecific virulence factors and Regulation of virulence-associated genes. The functional differences of virulence factors were compared among the groups at pathway level. The results showed (**Figures 10B–E**) that the gene abundance of four virulence factors, namely offensive virulence factors, defensive virulence factors, nonspecific virulence factors and regulation of virulence-associated genes, in the intestinal flora of APP/PS1 transgenic mice had a tendency to decrease compared with control mice, while the treatment of Qi-Fu-Yin had a tendency to increase them. There was a significant positive correlation (*P*<0.05) between grip strength and defensive virulence factors (**Figure 10F**), offensive virulence factors (**Figure 10G**), nonspecific virulence factors. (**Figure 10H**), and no significant correlation between grip strength and regulation of virulence-associated genes (**Figure 10I**). The above results indicated that defensive virulence factors, offensive virulence factors and nonspecific virulence factors may have a positive regulatory effect on grip strength in mice, and Qi-Fu-Yin may improve the motor coordination ability of aged mice by regulating related virulence factors.

#### The effect of Qi-Fu-Yin on the level of cytokines in the plasma of APP/PS1 transgenic mice

**Figure 11:**
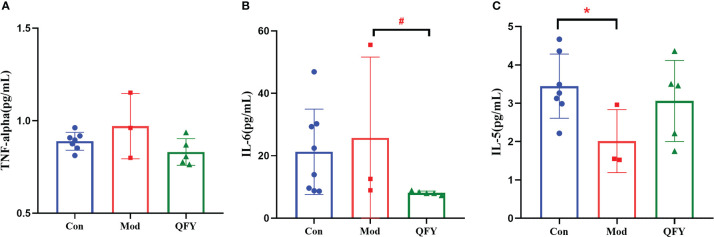
Effects of Qi-Fu-Yin on the level of cytokines in plasma of APP/PS1 transgenic mice. **(A)** TNF-α, **(B)** IL-6, **(C)** IL-5; Mean ± SD, n=3~8, ^*#^ *P*<0.05, unpaired Student`s *t*-test, Graphad Prism8.0.1 software.

After 195d administration of Qi-Fu-Yin in 11-month-old APP/PS1 transgenic mouse, the levels of pro-inflammatory factors IL-6 and TNF-α, anti-inflammatory factors IL-5 in the plasma of APP/PS1 transgenic mice were observed using the Luminex multi-factor assay. The results showed (**Figure 11**) that the plasma levels of TNF-α and IL-6 tended increase in APP/PS1 transgenic mice compared with the control group, while the administration of Qi-Fu-Yin reduced them, especially significant IL-6 (*P*<0.05) (**Figure 11B**). The level of anti-inflammatory factor IL-5 tended to decrease in APP/PS1 transgenic mice, while the administration of Qi-Fu-Yin had a tendency to increase. The above results suggested that administration of Qi-Fu-Yin might regulate immunity of aged APP/PS1 transgenic mice.

## Discussion

AD may involve the crossed nuclei of the hypothalamus and therefore may affect circadian and body temperature rhythms (Satlin A et al., 1995; Eggenberger et al., 2021). Cold tolerance was impaired in APP/PS1 transgenic mice at approximately 12 months old relative to WT (Kim et al., 2021). Memantine prevents glycogen depletion in the liver and lipid depletion in the adrenal glands of rats under cold stress conditions (Ferreira et al., 2011), thereby reducing heart stress due to cold stress (Meneghini et al., 2009). There have been reports of individuals feeling cold after administration of donepezil (Thiyagesh S and Spence, 2004). In this study, we found that Qi-Fu-Yin could significantly prolonged the survival days after cold stress.

It has been shown that no significant differences in tension and rotor-rod performance were observed between APP/PS-1 knock-in mouse model and the WT mice in any age group. However, rotor-rod performance is negatively correlated with weight (Webster et al., 2013). APP_695_SWE + PS1/M146L mice were impaired on the stationary beam at 5 and 16 months of age and on the suspended string at 16 months (Arendash et al., 2001). Nevertheless, motor coordination was not impaired in 7-month-old APPswe + PS1/E9 mice in rotational bar and grip strength tests (Lalonde et al., 2004). Our data indicated that the grip strength and motor coordination of rotarod were significantly reduced in APP/PS1 transgenic mice at about 16 months of age compared to the control group, while Qi-Fu-Yin significantly enhanced the ability of motor coordination in transgenic mice.

One study found that both 18-month-old APP/PS1 transgenic mice and wild-type mice failed to acquire associative learning of the reflex eyelid response, but only 3-month-old mice were able to fully acquire conditioned reflex blinks using a tracking paradigm, while 12-month-old wild-type and transgenic mice showed intermediate values (Arendash et al., 2001). The difference in escape latency of APP/PS1 transgenic mice in the Morris water maze in this study was not significant compared to the wild type, speculating that it may be due to the fact that the wild type animals are also in an old age with memory loss.

The gut microbiota is a large colony of bacteria in human. The increasing evidence showed that the intestinal flora was involved in a variety of human diseases and also had a regulatory function on central neurodegenerative pathologies (Jiang et al., 2017; Zhang et al., 2017). A growing body of clinical and experimental evidence suggests that the gut microbiota may contribute to aging and affect brain dysfunction (Jiang et al., 2017; Kim and Jazwinski, 2018). While such findings strongly suggested that the gut microbiota may impact a wide range of brain disorders including AD. Recently, a study found that brain amyloidosis in patients with cognitive impairment was associated with pro-inflammatory gut bacteria (Cattaneo et al., 2017). In addition, it has been shown that antibiotic-mediated disturbances in the gut microbiome modulate amyloid deposition in a mouse model of AD (Minter et al., 2016).

The study (Harach et al., 2017) showed that fecal samples from 1-, 3.5- and 8-months old APP/PS1 transgenic mice models were analyzed by bacterial 16S rRNA sequencing and revealed major age-related changes in the composition of the intestinal flora at both the phylum and genus levels compared to non-transgenic wild-type mice. The highest proportion of intestinal flora in APP/PS1 transgenic mice at 1-, 3.5- and 8-months old were all in the *Bacteroidetes*, which increased with the age of months. And the transgenic mice showed a significant increase in the abundance of *Bacteroidetes*, *Rikenellaceae* compared to the wild type at 8 months of age. During aging, *Rikenellaceae* increased in all groups, although proportions of *Firmicutes*/*Bacteroidetes* increased in APP/PS1 transgenic mice, whereas in WT mice decreased strongly (Bauerl et al., 2018). But the family *Erysipelotrichaceae* showed a tendency to increase with age in transgenic mice, and this bacterial family appears to be very immunogenic and highly coated by IgA in the gut (Palm Noah et al., 2014), it is abundant in inflammatory bowel disease (Schaubeck et al., 2016) and colorectal cancer (Chen et al., 2012). The above studies have shown that *Bacteroidetes* and *Rikenellaceae* are responsible for impaired pathophysiology and decreased cognitive function in AD, leading to early aging. Similarly, the reduced abundance of gut microbes in *Erysipelotrichaceae* has been associated with impairment of cognitive abilities, which may involve immunity. In our study, we found that *Bacteroidetes* had the highest proportion of intestinal flora in all three groups, followed by *Firmicutes*, which is consistent with what has been reported in the literature. We also found a significant increase in the abundance of *Bacteroidaceae*, *Bacteroides*, and *Rikenellaceae* in the intestinal flora of 17.5 months old APP/PS1 transgenic male mice, which is consistent with literature, meanwhile there was a trend of improvement after administration of Qi-Fu-Yin. However, in our study we found for the first time that the abundance of *Erysipelotrichaceae* was significantly reduced in APP/PS1 mice compared to non-transgenic mice. This provides future scope for the study of the host as an anti-aging microorganism.

Carbohydrate-active enzymes (CAZymes) are families of essential and structurally related enzymes, which catalyze the creation, modification, and degradation of glycosidic bonds in carbohydrates to maintain essentially all kingdoms of life (Benini, 2020). CAZymes play a key role in many biological processes underpinning human health and diseases (e.g., cancer, diabetes, AD, AIDS) and have thus emerged as important drug targets in the fight against pathogenesis (Williams, 2001; El Kaoutari et al., 2013; Montgomery et al., 2017). *N*-glycan is attached to nascent glycoproteins and is processed and matured by various glycosidases and glycosyltransferases during protein transport. Genetic and biochemical studies have demonstrated that alternations of the *N*-glycan structure play crucial roles in various physiological and pathological events including progression of cancer, diabetes, and AD (Nagae et al., 2020). Lysozyme aggregation in the presence of polyamines leads to nonneuropathic amyloidosis (Kabir et al., 2020). A meta-analysis supported that the methylenetetrahydrofolate reductase (MTHFR) C677T polymorphism was associated with an increased risk of AD (Rai, 2017). Clinical, epidemiological, and molecular biological evidence shows that disorders of glycolipid metabolism are positively associated with the risk of AD, and that disorders of central nervous system and peripheral glycolipid metabolism are present in the early stages of AD and accompany AD pathology (Peng et al., 2020; Kuehn, 2020). Qi-Fu-Yin contains a relatively rich variety of glycans, about 6% (Li et al., 2021). For the first time, we found that 8 of the top 10 active enzymes in the gut microbiota (GT2_Glycos_transf_2, GT4, GT41, GH2, CE1, CE10, CE3 and GH24) were significantly positively associated with grip strength. A Study has shown that Leukoencephalopathy caused by mutations in mitochondrial aspartyl-trna synthetase can manifest as severe brain atrophy and behavioral dysfunction (Nemeth et al., 2020). The above-mentioned active enzymes may have a positive regulatory effect on grip strength in mice, and this regulatory effect may be carried out through the relevant metabolic pathway. APP/PS1 transgenic mice may have increased the behavioral disorder by activating aspartyl-trna synthetase APP/PS1 transgenic mice may have increased the behavioral disorder by activating aspartyl-trna synthetase, while Qi-Fu-Yin can improve this impairment.

There is evidence that bacteria associated with periodontitis and their virulence factors can alter damage to the blood-brain barrier, and that aging and chronic exposure of the BBB to pathogens makes it permeable, which may contribute to AD (Kouki et al., 2022). The gene abundance of four virulence factors regulated by offensive virulence factor, defensive virulence factor, non-specific virulence factor, and virulence-related genes tended to decrease in APP/PS1 transgenic mice, while there was a trend of increase in the treatment with Qi-Fu-Yin. And the above virulence factors were positively correlated with grip strength. This study suggested that Qi-Fu-Yin may improve physical frailty of mice by regulating the above virulence factors for the first time.

A 20-25% reduction in cerebral glucose metabolism can be detected as early as the mild cognitive impairment (MCI) stage before the onset of AD and correlates with cognitive scores (Tournissac et al., 2021). Low brain glucose metabolism in AD patients leads to synaptic loss and neuronal death, and energy deficiency and neurotoxic protein accumulation exacerbate each other in a vicious cycle (Cunnane et al., 2020). Aβ plaque load was significantly increased in the hippocampus of PM2.5-exposed APP/PS1 mice compared to the respective filtered air (FA) control group (Sahu et al., 2021). Studies show that CD8 T cells infiltrate aged and AD brains and that brain CD8 T cells may directly contribute to neuronal dysfunction that regulates synaptic plasticity (Unger et al., 2020). Predictive models suggested that cognitive frailty was driven by dysregulation of multiple cellular processes, including genetic alterations, nutritional and lipid metabolism, and elevated levels of circulating pro-inflammatory proteins (Sargent et al., 2020). We compared the differences in KEGG functional enrichment and found that the abundance of all five KEGG genes in the intestinal flora of APP/PS1 transgenic mice for metabolism, environmental information processing, cellular processes, human diseases and biological systems tended to decrease compared to the control group, while after treatment of Qi-Fu-Yin tended to increase. Qi-Fu-Yin may play a role in anti-aging and cognitive improvement by regulating metabolism, cellular processes and other pathways.

In ELISA experiments (Yang et al., 2011), TNF-α and IL-6 in the brain tissue and serum of APP/PS1 transgenic mice were found to be elevated at 9 months of age compared with wild-type mice, and the differences were significant at 12 months of age and even more significant at 18 months of age. Compared to normal aging patients, concentrations of IL-5 were significantly higher in the superior frontal gyrus of patients with AD, followed by no significant difference in IL-6 (Tennakoon et al., 2022). In contrast, there were no significant differences in IL-5, IL-6, TNF-α in the plasma of tau P301S transgenic mice after 11 months of age (Sun et al., 2020). In this study, it was found that compared with the control group, 17.5-month-old APP/PS1 transgenic male mice showed a trend of elevated plasma TNF-α and IL-6 levels and a decreasing trend of anti-inflammatory factor IL-5 levels, which was reversed by the administration of Qi-Fu-Yin and significantly reduced IL-6 levels.

All the above analysis and discussion suggest that the mechanism by which Qi-Fu-Yin anti-aging in 17.5-month-old APP/PS1 transgenic mice may be through the regulation of intestinal flora diversity, species richness and the function of major active enzymes. This study combined aging and intestinal flora from behavioral to pathway levels, providing new ideas for further understanding the pathogenesis and mechanism of Qi-Fu-Yin against aging and AD. Unfortunately, the natural survival time of mice after treatment was not obtained in our study. In fact, on the premise of making it clear that Qi-Fu-Yin can anti-aging, further research on the functional mechanism of Qi-Fu-Yin anti-aging is the focus of our future research, especially those specific bacteria closely related to active enzymes, virulence factors, etc.

The major fundings on Qi-Fu-Yin for beneficial effect on aging of APP/PS1 transgenic mice in this study are as follows:

- Qi-Fu-Yin may have a positive effect on the spatial learning memory ability of aged APP/PS1 mice effect.
- Qi-Fu-Yin can significantly prolong the survival days after cold stress, indicating that Qi-Fu-Yin can weaken the cold stimulation, not only delaying but also reducing the death caused by cold stress.
- Grip strength and motor coordination are used to indicate physical frailty. Qi-Fu-Yin showed a tendency to enhance the grip strength of aged APP/PS1 transgenic mice. Qi-Fu-Yin significantly enhanced the ability of motor coordination in transgenic mice, and Qi-Fu-Yin may improve the motor coordination ability of aged mice by regulating related active enzymes and virulence factors
- Qi-Fu-Yin can regulate the bacteria such as *Bacteroidetes* and *Rikenellaceae* related to the pathophysiological and cognitive impairment in APP/PS1 transgenic mice, thus acting as an anti-aging and improving cognition.
- Qi-Fu-Yin improved the functional diversity of the major KEGG pathways, carbohydrate-active enzymes, and major virulence factors in the intestinal flora of APP/PS1 transgenic mice. Qi-Fu-Yin may play a role in anti-aging and improving cognitive ability by regulating metabolism, cellular processes and inflammatory factors.

## Data availability statement

The data presented in the study are deposited in the European Nucleotide Archive (ENA) repository (https://www.ebi.ac.uk/ena/browser/home), accession number PRJEB56657.

## Ethics statement

The animal study was reviewed and approved by Institute of Animal Care and Use Committee (IACUC) of Shandong University of Traditional Chinese Medicine.

## Author contributions

X-RC designed the study and modified the manuscript. Q-YX, carried out the specific studies. Q-YX, T-YY, X-LW, D-MQ and X-RC contributed to writing articles and modifying the manuscript. All authors contributed to the article and approved the submitted version.
