# Supplementary figures and images for: Effects of Qi-Fu-Yin on aging of APP/PS1 transgenic mice by regulating the intestinal microbiome
Source: Front Cell Infect Microbiol. 2023 Jan 13;12:1048513. doi: 10.3389/fcimb.2022.1048513 (PMC9880330; doi:10.3389/fcimb.2022.1048513)

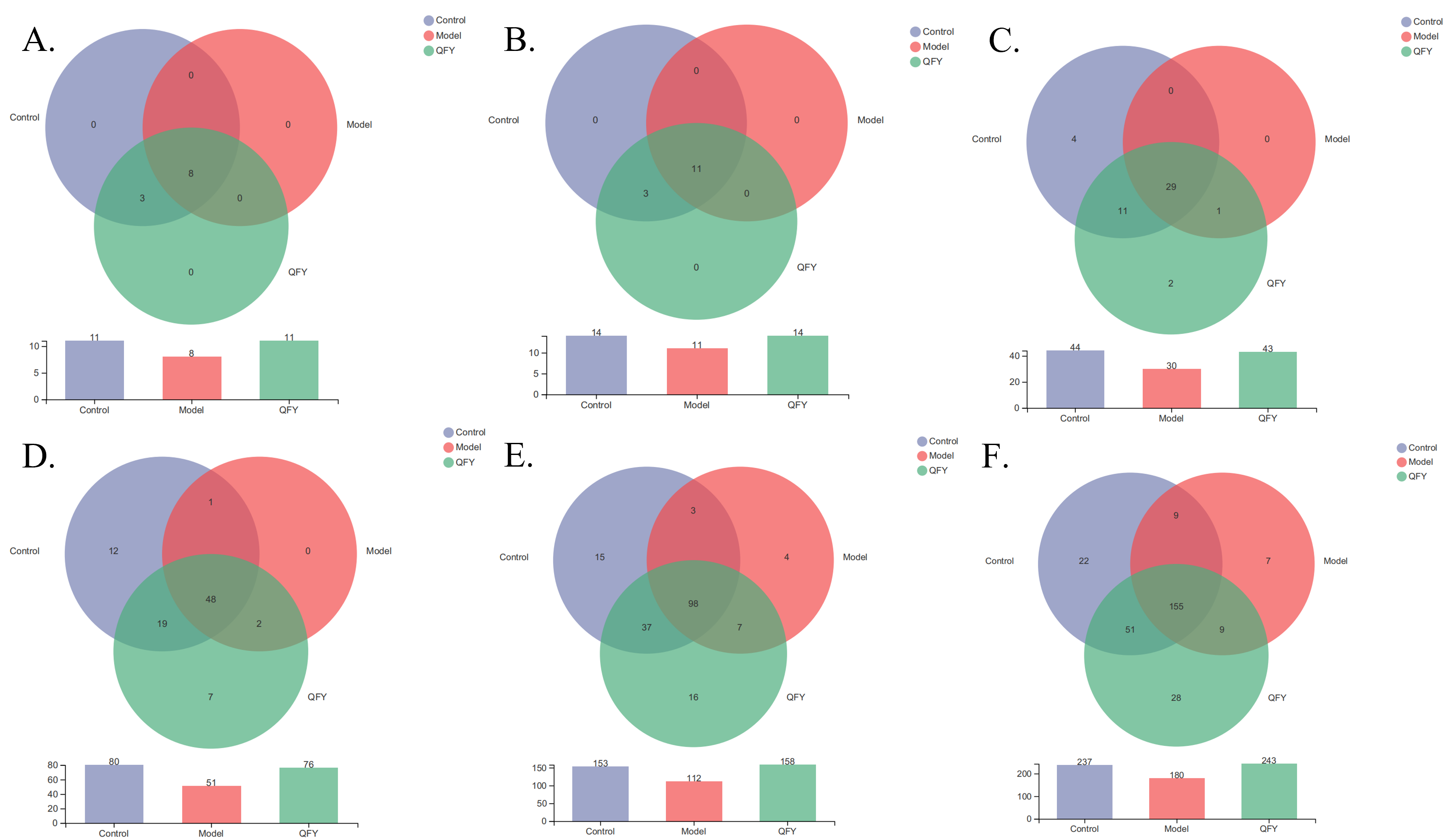

Supplement: Supplementary file 1 [file Image_1.tif]
